# Adaptation in the auditory system: an overview

**DOI:** 10.3389/fnint.2014.00019

**Published:** 2014-02-21

**Authors:** David Pérez-González, Manuel S. Malmierca

**Affiliations:** ^1^Auditory Neurophysiology Laboratory (Lab 1), Institute of Neuroscience of Castilla y León, University of SalamancaSalamanca, Spain; ^2^Department of Cell Biology and Pathology, Faculty of Medicine, University of SalamancaSalamanca, Spain

**Keywords:** auditory nerve, inferior colliculus, auditory cortex, stimulus-specific adaptation, mismatch negativity

## Abstract

The early stages of the auditory system need to preserve the timing information of sounds in order to extract the basic features of acoustic stimuli. At the same time, different processes of neuronal adaptation occur at several levels to further process the auditory information. For instance, auditory nerve fiber responses already experience adaptation of their firing rates, a type of response that can be found in many other auditory nuclei and may be useful for emphasizing the onset of the stimuli. However, it is at higher levels in the auditory hierarchy where more sophisticated types of neuronal processing take place. For example, stimulus-specific adaptation, where neurons show adaptation to frequent, repetitive stimuli, but maintain their responsiveness to stimuli with different physical characteristics, thus representing a distinct kind of processing that may play a role in change and deviance detection. In the auditory cortex, adaptation takes more elaborate forms, and contributes to the processing of complex sequences, auditory scene analysis and attention. Here we review the multiple types of adaptation that occur in the auditory system, which are part of the pool of resources that the neurons employ to process the auditory scene, and are critical to a proper understanding of the neuronal mechanisms that govern auditory perception.

## INTRODUCTION: THE AUDITORY SYSTEM NEEDS TO PRESERVE THE TIMING OF THE SIGNAL

The challenging task that the auditory system faces is to process naturally occurring sounds, so that they can be identified, characterized, and localized, in order to be able to respond accordingly and in a timely manner. A complication lies in the nature of sound, which consists of rapid variations of the pressure in an elastic medium, usually air for most mammals. One of the basic features of the components of all sounds is their frequency (or how fast the sound waves change) and the auditory brain must be able to extract it very precisely. The range of frequencies that each animal is sensitive to varies greatly. Humans typically can hear sounds from 20 Hz to 20 kHz. Some animals have good low frequency hearing, similar to humans, like the guinea pig (Heffner et al., 1971), but other animals can hear much higher frequencies. For instance, mice can hear sounds over 80 kHz (Heffner and Masterton, 1980) and some bats up to 120 kHz (Koay et al., 1997). In order to process these very rapid variations of the signal, the auditory system requires fast and reliable responses from its elements. Timing information is also essential for the localization of sounds, since it requires a precise encoding of the time at which sounds arrive at each ear. The detection of the minimum change for sound localization in the horizontal plane in humans requires comparing the arrival time at both ears with a precision of a few microseconds (Hafter et al., 1979; Kollmeier et al., 2008).

The timing of action potentials, conveyed with the precision of microseconds, carries acoustic information in all higher vertebrates. For instance, responses of low-frequency auditory nerve fibers are locked to a particular phase of the stimulus waveform (Kiang et al., 1965; Johnson, 1980; Palmer and Russell, 1986), and thus carry a temporal code for sound frequency. The requirement of a precise and faithful transmission of timing information has given rise to the development of certain cellular specializations. The auditory nerve fibers that innervate the anterior ventral cochlear nucleus in mammals have large, specialized calyceal endings, also known as endbulbs, that surround the soma of the target neuron (for a review, see Ryugo and Parks, 2003). In other cells, the synchronization of their responses is enhanced thanks to the convergence of a few auditory nerve fibers through large endbulbs (Ryugo and Sento, 1991; Joris et al., 1994).

This faithful encoding of auditory information is maintained along the ascending auditory pathway up to the auditory cortex (AC), whose neurons are capable of maintaining millisecond precision in the encoding of auditory stimuli (Kayser et al., 2010). But, while the auditory system is so deeply dependent on timing, there are still many instances where adaptation processes take place. Adaptation, as we will consider in this paper, consists on a decrease of the response of a neuron or population or neurons during stimulation, and may manifest itself in several ways. For the sake of simplicity and descriptive purposes, here we differentiate adaptation from habituation, which is commonly used in reference to perceptual and behavioral phenomena, and is more closely related to learning processes. In this review, we will focus on the multiple forms that neuronal adaptation takes through the auditory system.

## ADAPTATION OF THE AUDITORY NERVE FIBERS

**FIGURE 1:**
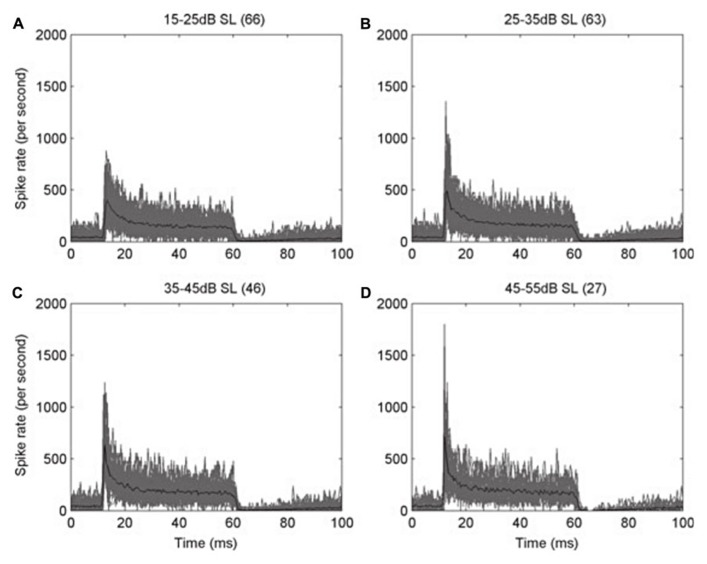
**Peri-stimulus time histograms (PSTH) of the response of auditory nerve fibers of the ferret.** The action potentials of auditory nerve fibers with high characteristic frequency (CF > 1.5 kHz) were recorded during the presentation of a 50 ms pure tone at the CF of each fiber, and then the spike times from all the fibers were pooled. **(A–D)** Each panel shows the overplotted response (gray) from the number of fibers in parentheses, at different levels above their threshold, as indicated on the top of each panel. The mean values are plotted in black. Note how the initially high response rate decreases rapidly to a steady state, in an example of spike-frequency adaptation. Bin width is 0.5 ms. Reproduced from Sumner and Palmer (2012).

Adaptation in the auditory system occurs as early as in the auditory nerve fibers. As has been classically described in other sensory neurons (Adrian, 1926; Adrian and Zotterman, 1926a,b), auditory nerve fibers (**Figure 1**) in all studied species show adaptation (e.g., Nomoto et al., 1964; Kiang et al., 1965; Feng et al., 1991). It takes the form of a higher instantaneous firing rate when a stimulus is switched on, slowing to a lower steady-state rate after a few tens of milliseconds (e.g., **Figure 1**; Sumner and Palmer, 2012). This particular type of adaptation is also known as spike-frequency adaptation, in which a neuron’s response to a steady-state stimulus is not maintained at its initially high rate of spiking but instead declines over time to a lower, adapted rate (**Figure 1**). This is a common feature of many sensory neurons (Hille, 1992). This type of response is the origin of the classic “primary” response of auditory nerve fibers, a well-described example of adaptation in the peripheral auditory pathway (Westerman and Smith, 1984; Yates et al., 1985). It is interesting to note that the adaptation is stronger in high frequency fibers than in low frequency fibers (Sumner and Palmer, 2012), especially since low frequency fibers are the ones that show phase locking. This way, the timing information carried by phase locking fibers is preserved. One possible role for adaptation in the auditory system lies in determining the sensitivity of auditory neurons to the stimulus context. The rapid adaptation in auditory nerve fiber responses (Yates et al., 1985; Westerman and Smith, 1987), and the rapid recovery from adaptation (Yates et al., 1983), suggests that the time course of adaptation in the peripheral nerve fibers might dominate the time course of adaptation in higher centers, unless it is somehow filtered out by neurons at subsequent stages. Indeed, adaptation in these early stages of the auditory pathway may have important implications in the processing of auditory cues at higher centers. In crickets, Givois and Pollack (2000) found that the receptors ipsilateral to the sound source became more adapted than the contralateral ones, since the sound arrives with higher intensity to the ipsilateral side. The different amounts of adaptation produced an imbalance in the interaural difference in response strength, increasing the difficulty of using the interaural level difference as a cue for sound localization. In that situation, they found that the neuronal response latency was more stable, and thus the interaural latency difference was a more reliable cue for sound source localization.

A phenomenon potentially related to adaptation in the auditory nerve is forward masking. It consists in the elevation in the threshold of a signal caused by the presence of a masker sound preceding it in time, and has been the subject of intense study over a number of decades (Harris and Dallos, 1979). Since the preceding, masking sounds caused an apparent reduction of the neuronal responses, adaptation in the auditory nerve has been proposed as a candidate for the neural site of forward masking (Smith, 1977, 1979). However, some studies suggest that forward masking is better explained by temporal integration models, rather than adaptation (Oxenham, 2001), and so this issue is still a matter of debate.

## ADAPTATION BECOMES MORE DIVERSE ALONG THE AUDITORY HIERARCHY

Firing rate adaptation has been also found in other brainstem nuclei. For instance, Finlayson and Adam (1997) studied short-term adaptation in the superior olivary complex, a group of auditory brainstem nuclei that are involved most notably in the extraction of binaural cues for sound source localization. They found that acoustic stimulation results in rapid and prolonged adaptation in monaurally driven excitation and inhibition of these neurons. For neurons where both the ipsilateral and contralateral inputs are equally affected by adaptation, the effect on localization accuracy is very small. On the other hand, they found that in some neurons the adaptation from ipsilateral and contralateral stimulation is unbalanced, which may affect the coding of localization cues. Finlayson and Adam (1997) conclude that this unbalanced adaptation should cause a poor localization performance by these neurons in noisy conditions.

As we examine higher auditory centers, we can find more complex types of adaptation. The inferior colliculus (IC), the mammalian midbrain auditory nucleus, has received quite considerable attention lately. The IC is a mandatory relay for almost all the ascending auditory information en route to the thalamus and cortex. It receives ascending inputs from most of the lower brainstem nuclei and descending inputs from the cortex (Malmierca and Hackett, 2010; Malmierca and Ryugo, 2011). Therefore, there is no doubt that the IC is strategically located and able to combine and process the information extracted by the previous auditory pathways, so it is not surprising to find more developed neuronal responses.

Processes of spike-frequency adaptation have been described in the IC (Ingham and McAlpine, 2004), in neurons sensitive to interaural phase disparities. In these neurons, it was possible to use a binaural stimulation paradigm that allowed separation of the adaptation of the binaural neurons from that happening at lower monaural levels, such as the auditory nerve fibers. This study revealed that these IC neurons had adaptation dynamics that were rather slow, compared with those calculated for the auditory nerve fibers (Yates et al., 1983, 1985; Westerman and Smith, 1987). The different time constants indicate that the adaptation found in the IC is different from that found in the auditory nerve, and moreover, it is not just inherited from the lower levels.

A different type of adaptation found in the IC is the adaptation of the population coding to stimulus statistics. Dean et al. (2005) studied this type of adaptation regarding the processing of sound level. Mammals can hear sounds extending over an immense range of sound levels with remarkable accuracy. How auditory neurons code sound level over such an extensive range is unclear, since firing rates of individual neurons increase with sound level over only a very limited portion of the full range of hearing. Using stimuli whose intensity changed in a probabilistic way, Dean et al. (2005) found that neural responses were rapidly adjusted by adaptation, in a manner that improved the coding of the most probable sound levels by the neural population.

**FIGURE 2:**
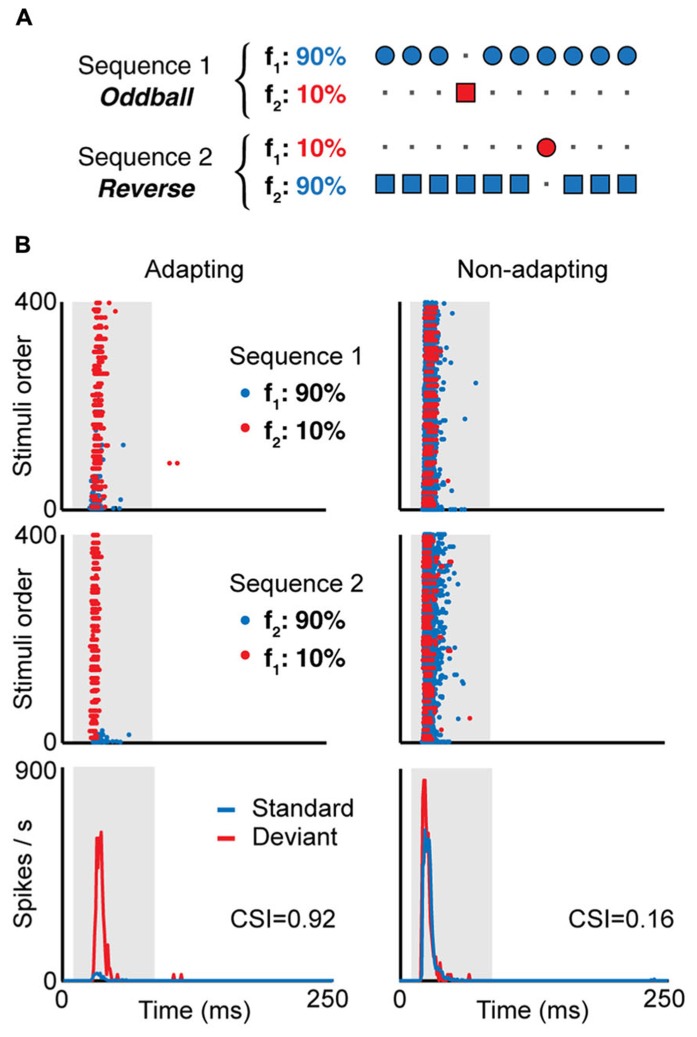
**(A) In the oddball paradigm, a low probability stimulus (f_2_, red, “deviant”) is embedded in a train of high probability stimuli (f_1_, blue, “standard”).** To compensate the responses to the different physical stimuli f_1_ and f_2_, a second sequence is presented where the probability of each stimulus is reversed. Examples of the responses of two neurons recorded using this paradigm is shown in **(B)**, an IC neuron showing SSA (adapting, left) and another not showing SSA (non-adapting, right). Here, f_1_ and f_2_ are pure tones of different frequencies, and the frequency difference is the same for both neurons. The top and middle panels in **(B)** show the dot raster in response to sequence 1 (top) and sequence 2 (middle), where the blue dots represent spikes in response to the standard and the red dots the response to the deviant. In the adapting neuron, the response to the standard stimulus decays after the first presentations, while the response to the deviant stimulus remains constant, as a typical example of stimulus-specific adaptation. The bottom panels show the PSTH for the responses to the standard and deviant stimuli, combining the spikes for both stimuli at the same probability. The value of the common SSA index (CSI) is shown for each neuron; CSI values close to one indicate strong SSA while values close to zero indicate weak SSA. Adapted from Duque et al. (2012).

**FIGURE 3:**
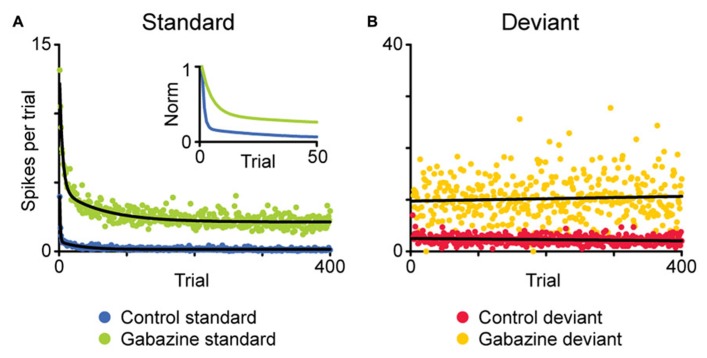
**Average time course of the adaptation of a population of neurons showing SSA in the IC.** Using an oddball paradigm, the spike count after each stimulus presentation is plotted based on whether the given stimulus was standard **(A)** or deviant **(B)**. In the baseline condition, the responses to the standard stimulus adapt rapidly after a few presentations **(A**, blue), while the responses to the deviant stimuli do not experience adaptation **(B**, red). The application of gabazine increases the responses to both stimuli (green, standard; yellow, deviant), but the dynamics of adaptation remain similar. The inset in **(A)** shows the normalized response for the standard stimuli, to better compare both time courses of adaptation. Adapted from Pérez-González et al. (2012).

Neurons in the IC also show stimulus-specific adaptation (SSA, **Figure 2**). These neurons reduce their responses to a stimulus that is presented repeatedly, but when a novel sound is presented, the same neurons are able to overcome the adaptation and response quickly and vigorously (e.g., **Figures 2** and **3**; Pérez-González et al., 2005; Malmierca et al., 2009). An increase in response strength with the presentation of a stimulus change can be explained by a release from adaption, but particularly when measured in single neurons, it indicates that the underlying adaptation processes are stimulus- (or feature-) specific, thus enabling the system to differentiate stimuli not by their absolute dimensions but by their relative attributes across space and time (Moore, 2003). For these and other reasons, SSA has been proposed to play a role in the attention and the detection of auditory deviance, change and novel stimuli. While SSA was first described in the AC (Ulanovsky et al., 2003), the IC is the lowest nucleus where it is present (Lumani and Zhang, 2010; Zhao et al., 2011; Ayala and Malmierca, 2013; Ayala et al., 2013); it has also been found in the auditory thalamus (Anderson et al., 2009; Antunes et al., 2010; Bäuerle et al., 2011; Antunes and Malmierca, 2014; Duque et al., 2014). The different studies have noted that SSA is stronger in the non-lemniscal divisions of the subcortical auditory nuclei. For instance, it is more prominent in the rostral, dorsal and lateral subdivisions of the IC (Duque et al., 2012), and also in the medial division of the geniculate body (Antunes et al., 2010). On the other hand, in the lemniscal regions, like the central nucleus of the IC and the ventral nucleus of the geniculate body, fewer neurons show SSA, and it is weaker. It seems that SSA is generated *de novo* in each level, and it is not clear that SSA generated in one nucleus propagates to the other, either in a bottom-up or a top-bottom fashion (Antunes and Malmierca, 2011, 2014; Anderson and Malmierca, 2013).

**FIGURE 4:**
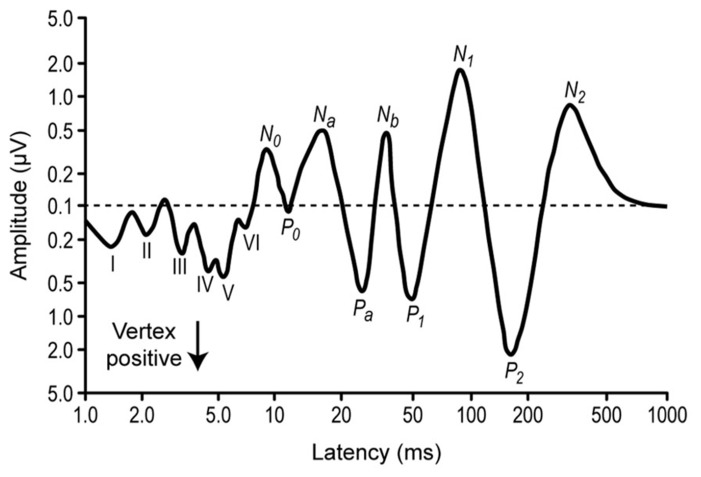
**Diagrammatic representation of the auditory evoked potential components.** Average from several subjects. The stimuli were clicks presented monaurally, and the EEG was recorded from a electrode in the vertex position and a reference at the mastoid. The components from the first 10 ms (I–VI) correspond to the auditory brainstem responses (ABR), around 20–50 ms correspond to the middle latency responses, and 150–250 ms correspond to the activation of the frontal cortex. Note the logarithmic scales. Redrawn from Picton et al. (1974).

**FIGURE 5:**
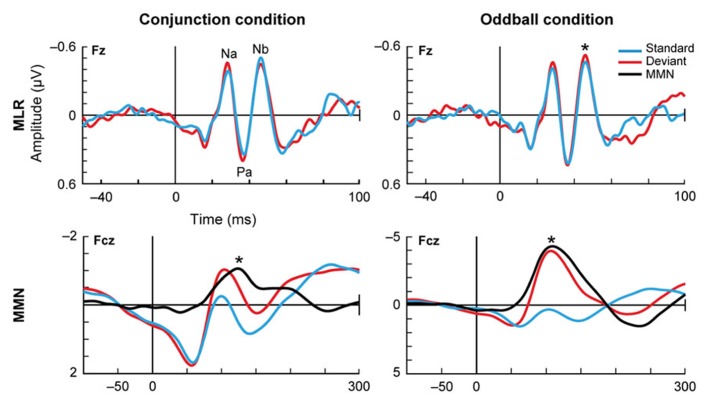
**Deviance detection in humans.** Althen et al. (2013) measured the auditory evoked potentials in response to paradigms of different complexity: the simple deviance detection of the frequency oddball paradigm (right) and a more complex “conjunction” paradigm (left), where the standard stimuli consisted of certain frequency-location combinations and the deviants broke that correspondence, combining one frequency with the opposite location. This figure shows the grand-average for 18 subjects, with the data filtered either for the middle latency range (top plots) or the long latency range (lower plots). In the middle latency range, there is a reduced response to the standards compared to the deviants, but only in the oddball condition. In contrast, in the long latency range this reduction of the standards occurs for both conditions. These findings suggest a hierarchy in the detection of deviance. Asterisks indicate significant differences. Reproduced from Althen et al. (2013).

While SSA in the IC was originally described from the neuronal responses using extracellular recordings in animals (**Figures 2** and **3**), including local field potentials (von der Behrens et al., 2009; Patel et al., 2012), a correlate has been found measuring ERP in the human auditory brainstem (**Figure 4**) using the frequency-following response (Slabu et al., 2012), showing that the human IC is able to detect a novel acoustic event occurring among a series of repetitive ones. This is supported by the attenuated human brainstem response (see **Figures 4** and **5**) to a stimulus occurring with a low probability compared with that elicited by the same physical stimulus presented with much higher probability. Their findings suggest that the human auditory brainstem is able to encode acoustic regularities in a memory trace and to detect deviant events based on a comparison process between the current auditory input and the recent auditory history. These results are in agreement with previous studies using the frequency-following response, that showed that the human auditory brainstem encodes stimulus statistics over multiple time scales (Chandrasekaran et al., 2009; Skoe and Kraus, 2010a, b). Similar results have also been observed for cortical neurons (Ulanovsky et al., 2004) and human cortical-evoked potentials (Costa-Faidella et al., 2011). These and other studies have shown the presence of different types of adaptation and deviance-related activity over several time ranges of the auditory event-related potentials and strongly support the idea of a hierarchically organized system devoted to auditory deviance detection (Grimm et al., 2011; Grimm and Escera, 2012).

The mechanism for SSA is still under investigation, and several options have been put to the test. One of the possibilities under consideration is that SSA emerges from the intrinsic characteristics of the cell, such as the membrane properties. Duque et al. (2012) found that the strength of SSA in IC neurons is not constant within their receptive fields; instead it varies systematically in each neuron, being stronger in the high frequency region as well as near the threshold. If the origin of SSA were based in the intrinsic properties of the cell, its strength should be more uniform within the receptive field, so these results contradict this possibility. Another possible mechanism would be based on the effect of synaptic inhibition, but again, it seems to be unlikely. The pharmacological manipulation of GABA_A_ receptors, in the IC (Pérez-González et al., 2012; Pérez-González and Malmierca, 2012) as well as in the auditory thalamus (Duque et al., 2014), has shown that, while not involved in the generation of SSA, the inhibitory inputs could modulate its strength, acting as a gain control mechanism, in some instances similar to the iceberg effect (**Figure 3**). Instead, a likely mechanism for SSA is one based on the differential adaptation of the inputs to the cell showing SSA (May and Tiitinen, 2010). Comparing multiple stimulus presentation paradigms, Taaseh et al. (2011) proposed that in the AC, SSA is mediated by “adaptation channels,” that would span the receptive field of the neuron. In this model, SSA would emerge from the differential adaptation of the channels, as determined by the frequency of the stimuli and their separation. However, other complementary explanations may be needed to fully explain the formation of SSA.

## ADAPTATION IN THE AUDITORY CORTEX

**FIGURE 6:**
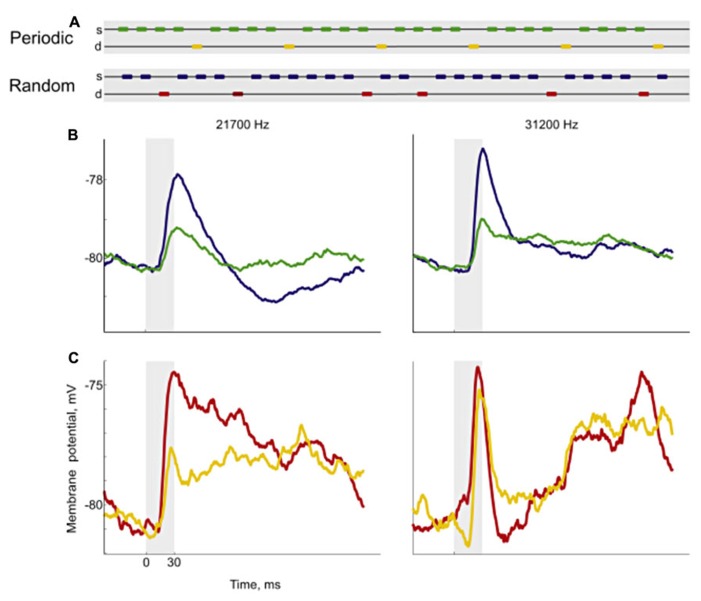
**Responses to periodic and random sequences.** Using variations of the oddball paradigm, Yaron et al. (2012) developed sequences with either periodic or random deviants **(A)**. For each neuron, they chose a pair of frequencies and constructed sequences with the same deviant probability where the only difference was whether the position of the deviant was periodic (yellow marks) or random (red marks). Then they recorded the neuronal responses of cortical neurons, measured from their membrane potentials **(B,C)**. The left plots show the responses for one of the frequencies and the right plots for the other. For the standard condition, the responses were smaller in the periodic sequence (green) than in the random sequence (blue). In the case of the deviant, the responses to the periodic (yellow) and the random sequence (red) were different for one frequency (left) but not for the other. These results show that cells in the auditory cortex are able to code complex regularities. Reproduced from Yaron et al. (2012).

Because of its complex organization and connectivity, including the fact that it is receiving the information that has been extensively processed by all the previous nuclei in the pathway, it is not surprising to find the most numerous types of adaptation processes occur in the AC. Ter-Mikaelian et al. (2007) found that the responses to continuous stimuli adapted with faster kinetics in the primary AC than in the IC, indicating that different temporal filters operate in the different nuclei, which may influence the coding of information in each center. However, in some instances, adaptation processes are quite similar to the counterparts previously described in earlier nuclei. For instance, the SSA found in the AC is quite similar to that found in the IC (Malmierca et al., 2009; Duque et al., 2012) and auditory thalamus (Antunes et al., 2010; Duque et al., 2014), at least in its basic appearance [rat: (Lazar and Metherate, 2003; Szymanski et al., 2009; Farley et al., 2010; Taaseh et al., 2011); cat: (Ulanovsky et al., 2003, 2004)]. However, the range of parameters eliciting SSA seems to be unique for each center, probably reflecting the particular processing capabilities of the neurons. For instance, SSA is elicited by faster repetition rates in the IC (Malmierca et al., 2009) and the thalamus (Antunes et al., 2010) than in the cortex (e.g., Taaseh et al., 2011). On the other hand, the ability to produce SSA with slow repetition rates is not a characteristic exclusive to the cortex, since SSA has been demonstrated in the IC with similar or even longer interstimulus intervals (Zhao et al., 2011; Ayala and Malmierca, 2013). It is also likely that the cortical neurons are capable of processing more complex sequences than those in lower nuclei (e.g., **Figure 6**; Yaron et al., 2012), and indeed, some results suggest that the processing of sequences is hierarchically structured, with higher centers able to process more complex sequences (Althen et al., 2013; Escera and Malmierca, 2014). However, related studies of subcortical structures are still scarce. Moreover, strong SSA has been reported in the primary AC, being the first lemniscal structure where it has been found, in contrast to the IC and thalamus, where SSA is more prominent in the non-lemniscal subdivisions. The different characteristics of SSA in cortical and subcortical nuclei invites caution when combining the studies performed in each of them. While they probably share some of the mechanisms proposed to create SSA, as explained earlier, each center may add particular conditions that may not extrapolate to the other. For instance, a study demonstrated an analog of SSA in cultured networks of cortical neurons. Eytan et al. (2003) used a paradigm of electrical stimulation similar to the oddball design, and found a depression in the responses to the standard and an increased response to the deviant. Furthermore, this selective enhancement of responses was abolished by blocking GABAergic inhibitory transmission using bicuculline. They proposed that the enhancement of the response to the deviant stimuli was caused by an adaptation of the inhibition, since both standard and deviant stimuli activated the inhibitory circuits. While this is a plausible explanation for cultured cortical neurons, it is unlikely to explain SSA in the IC *in vivo.* We have previously mentioned GABA_ A_-mediated inhibition (**Figure 3**) does not have such effect in the IC (Pérez-González et al., 2012), and hence is another example of the differences in SSA between centers.

**FIGURE 7:**
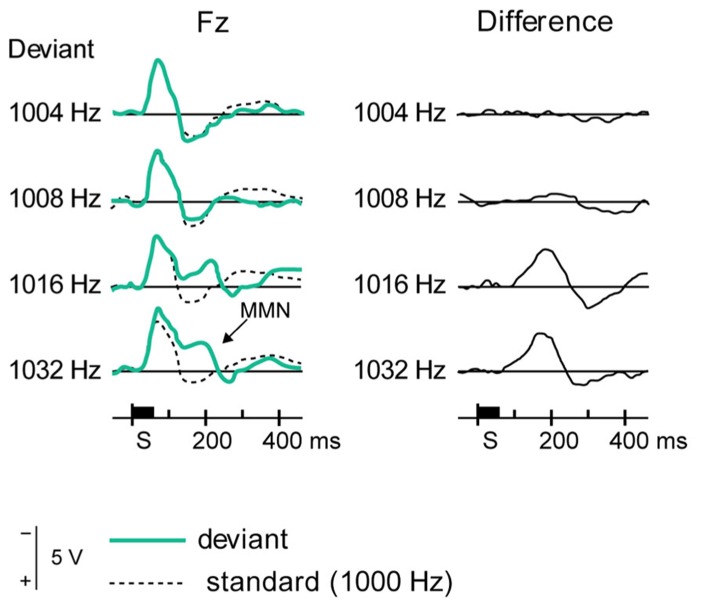
**Eliciting MMN.** The event-related potentials, averaged from several subjects (left panels), show differential responses to standard stimuli (dotted lines) and several deviant stimuli (green lines), as recorded from a frontal electrode. The difference (right panels, solid black lines) shows a deflection at around 200 ms, which constitutes MMN. Reproduced from Näätänen et al. (2007).

However, most examples of adaptation in the AC have been shown by the recordings of evoked potentials, since this part of the brain is very well suited for this technique. For the same reasons, the cortex is the center where most studies have been carried in humans. Using this technique, adaptation is expressed by reduced amplitude of the evoked response to repeated stimulation (Megela and Teyler, 1979). Adaptation in the cortex seems to be involved with the processes of deviance or change detection. These processes have been studied through experiments that analyze a component of evoked potentials known as mismatch negativity (MMN, **Figure 7**). MMN is evoked by a passive oddball paradigm, where a deviant stimulus is embedded in a train of common, high probability stimuli. MMN is the comparison of the responses to the deviant and common stimuli, resulting in a wave that peaks 150–250 ms after the stimulus onset (Näätänen et al., 1978, 2007). In this context, adaptation would be involved in the reduction of the response to the repetitive, high probability stimuli. MMN has been proposed to reflect the comparison of the deviant stimulus with the neuronal trace of the previous stimuli, and it even could be considered some kind of “primitive intelligence” (Näätänen et al., 2001, 2007). One of the characteristics of this change detection system is that it is pre-attentive and automatic, not requiring conscious processing, as indicated by the fact that it persists during sleep and under anesthesia (King et al., 1995; Atienza et al., 2001, 2002). It has been proposed to rely upon a concatenated set of basic adaptation mechanisms and what Bregman referred to as a “bottom-up” or “primitive” grouping (Bregman, 1990; Fritz et al., 2007). The change detection system could be involved in the process of auditory attention (Fritz et al., 2007) or auditory stream segregation (Sussman et al., 2005).

The effects of adaptation in the AC are various. Condon and Weinberger (1991) showed that the repetitive presentation of a stimulus caused long-term frequency-specific changes in the receptive fields of cortical auditory neurons, indicating that adaptation produced a change in the processing of frequency information rather than a general reduction in responsivity. These plastic changes in the AC may be mediated by noradrenergic inputs. The locus coeruleus is a prominent source of noradrenaline which innervates widespread brain regions, including the tectum, the thalamus and the cortex (Sara, 2009). Edeline et al. (2011) used electrical stimulation of the locus coeruleus paired with auditory stimulation to produce plastic changes in the receptive fields of neurons in the AC and thalamus. In fact, it has been shown that the same neurons of the locus coeruleus experience adaptation to auditory stimuli, among others (Herve-Minvielle and Sara, 1995; Vankov et al., 1995). Cholinergic inputs are another possible candidate for modulating adaptation in the cortex, since its role in processes of cortical plasticity has been shown previously (Metherate and Weinberger, 1989; Kilgard and Merzenich, 1998), but the extent of this possibility awaits future experiments. Acetylcholine is also a tentative modulator of adaptation phenomena in subcortical structures, since it has been shown that cholinergic nuclei in the tegmentum innervate the IC and the auditory thalamus (Motts and Schofield, 2011). Deouell et al. (2007) showed using fMRI that a region in the human medial planum temporale is sensitive to background auditory spatial changes, even when subjects are not engaged in a spatial localization task, and in fact attend the visual modality. During such times, this area responded to rare location shifts, and even more so when spatial variation increased, consistent with spatially selective adaptation.

## RELEVANCE OF ADAPTATION IN THE AUDITORY SYSTEM

One of the earliest roles assigned to cortical adaptation is the protection against cortical overstimulation (Megela and Teyler, 1979). This way, the reduction of neuronal activity during repetitive stimulation would have a protective effect, avoiding an overload of the processing systems. As we have mentioned previously, adaptation could also have a role in the detection of auditory change and novelty, as revealed by the experiments on SSA (e.g., Ulanovsky et al., 2003; Malmierca et al., 2009) and MMN (e.g., Escera et al., 1998; Näätänen et al., 2005), as well as auditory attention (Fritz et al., 2007).

Recently, adaptation has been proposed as a way of achieving an efficient coding of the incoming information (Wark et al., 2007). This would suggest that adaptation in stimulus encoding would be sensitive to the variations in stimulus statistics. By adapting to the current distribution of the stimuli, their values and changes could be represented more precisely. This view is supported by findings like that neurons in the auditory midbrain adjust their responses to the statistics of sound level distributions (Dean et al., 2005), improving the accuracy of the neuronal population code and extending the range of sound levels that can be accurately encoded. This is probably a widespread function for adaptation, since even in grasshoppers it has been found that the recognition of temporal patterns is improved by neuronal adaptation (Ronacher and Hennig, 2004). A number of studies, most notably in the visual system, have suggested a role for adaptation of excitation in scaling neural output to take account of, for example, stimulus variance (Brenner et al., 2000; Fairhall et al., 2001). It is also well described that complex cells of the visual cortex adapt to the local contrast (Ohzawa et al., 1982; Laughlin, 1989; Carandini and Ferster, 1997), the effect being to position a neuron’s dynamic range of discharge rates over the relevant range of contrasts.

But not all the response decrements are necessarily related to adaptation. Studying the decrement of the N1 auditory event-related potential (**Figure 4**) with stimulus repetition, Budd et al. (1998) argue that this decrement is based on the separate refractory periods or recovery cycle processes of at least two neural generators contributing to activity in the N1 peak latency range, rather than on an adaptation process. An important feature of the N1 peak of the auditory event-related potential is its systematic reduction in amplitude when the eliciting stimulus is repeated. A major psychophysiological issue regarding the functional nature of N1 amplitude decrement has been the extent to which this response decrement reflects a psychologically relevant process or a more basic neurophysiological process. One method of distinguishing between the distinct processes of adaptation and refractoriness is that amplitude reductions caused by refractoriness should stabilize immediately after repetition of a stimulus while adaptation could entail a more progressive decline in responsiveness (Picton et al., 1976).

## CONCLUSION

Adaptation phenomena are widespread in the auditory system, different to habituation, and they appear in multiple forms. Spike-frequency adaptation is already present in the auditory nerve fibers, while nevertheless preserving the timing information. The responses of the auditory fibers, despite adaptation are able to carry enough timing information, like the onset and duration of sounds. It is noteworthy to note that phase-locking fibers, which would carry additional timing information, seem to experience weaker adaptation (Sumner and Palmer, 2012). This early balance of adaptation and timing information must be appropriate to allow the processing of basic acoustic features in the brainstem nuclei, such as sound location. It is interesting the fact that other, more elaborate types of adaptation appear in higher levels, maybe because the basic timing information is no longer required. These other types of adaptation could contribute to further processing of the information stream. For instance, in the midbrain the SSA contributes to change and deviance detection. At higher levels, adaptation allows neurons to process more intricate characteristics of the auditory environment, such as abstract relations, complex sequences and regularities, and eventually to contribute to processes like auditory attention and stream segregation.

## Conflict of Interest Statement

The authors declare that the research was conducted in the absence of any commercial or financial relationships that could be construed as a potential conflict of interest.
